# Predictability of postural sway: unraveling the impact of simulated somatosensory deficits using a rambling-trembling approach

**DOI:** 10.3389/fbioe.2025.1572309

**Published:** 2025-09-05

**Authors:** Eryn D. Gerber, Chun-Kai Huang, Camilo Giraldo, Paris Nichols, Carl W. Luchies

**Affiliations:** ^1^ Biodynamics Research Laboratory, University of Kansas, Lawrence, KS, United States; ^2^ Department of Physical Therapy, Rehabilitation Science, and Athletic Training, University of Kansas Medical Center, Kansas City, KS, United States; ^3^ Department of Engineering, Messiah University, Mechanicsburg, PA, United States; ^4^ Department of Mechanical Engineering, University of Kansas, Lawrence, KS, United States

**Keywords:** center of pressure, postural control, rambling-trembling, falls, nonlinear analysis

## Abstract

One of the primary contributors to falls in older adults is somatosensory degeneration. A method of center-of-pressure (COP) analysis, rambling-trembling (RM-TR) decomposition, has the potential to significantly improve balance deficit detection. However, its ability to capture sensation-driven changes to postural sway is not well understood. Therefore, the objective of this study is to quantify the effects of progressive simulated somatosensory deficit on COP, RM and TR time series. Fifty-one healthy adults (aged 22.10 ± 1.88 years) completed three 60-s double-limb, quiet standing trials with eyes closed for each randomly-ordered foam thickness condition (no foam, 1/8″, 1/4″, 1/2″, and 1″). Foot-floor kinetic data was collected at 100 Hz using two 6-axis force plates and a 16-bit A/D acquisition system. The data were filtered with a 2nd-order 10 Hz low-pass Butterworth filter and used to calculate COP, RM and TR time series. Range, root-mean-square (RMS), and sample entropy (SampEn) were calculated for each time series. Repeated measures analyses of variance, with α = 0.05, were conducted to compare foam condition for each measure (range, RMS, and SampEn). Results showed range and RMS increased with foam thickness; thicker foams (F3–F4) produced larger increases than thinner foams (F1–F2), with more prominent effects in the AP than ML direction. SampEn decreased as foam thickness increased, but not for all comparisons or measures. TR consistently showed the greatest SampEn values compared with COP and RM. Our findings suggest that RM-TR decomposition can isolate distinct biomechanical contributions to postural sway, each influenced independently by somatosensation. Future work should continue to explore the utility of RM-TR decomposition, particularly in aging populations, to advance our understanding of sensory contributions to postural control and assess its viability as a clinical assessment tool.

## 1 Introduction

In the United States, 28.7% of adults aged 65 or older experience at least one fall every year, amounting to an estimated 29 million falls (Bergen et al., 2016). Efforts have been made to identify fall risk in older adults, but a significant portion of the population remains unknowingly vulnerable to falls due to the limitations of current clinical assessment techniques. In fact, there remains an estimated 8%–12% risk of fall over the course of the year for individuals with no identifiable risk factors (Chang and Ganz, 2007; Robbins et al., 1989).

As we age, our bodies experience a multitude of changes that ultimately result in a progressive decline in physiological function (Francis et al., 2017; Henry and Baudry, 2019). Among these changes is somatosensory loss. Somatosensation allows us to perceive environmental and proprioceptive cues that inform postural adjustments, maintain balance, and move safely and efficiently (Inglis et al., 1994; Simoneau et al., 1995). As functionality of this system declines, the risk of severe falls, injury, and death increase substantially (Ambrose et al., 2013; Shaffer and Harrison, 2007).

To address the prevalence of falls in older adults and remedy the limitations of existing clinical assessment techniques, many researchers have attempted to quantify balance by measuring movement of the body’s center-of-pressure (COP) (Winter et al., 1990). COP analysis has been used extensively in the study of human balance, including in investigations of aging and disease, but is limited in its clinical implications due to the uncertainty in its link to the underlying physiological mechanisms that dictate it Collins and De Luca (1993). Because of this disconnect, many of these analyses lack the reliability and sensitivity, on a patient-by-patient basis, to capture age-related balance changes, especially those that occur prior to the first fall (Lin et al., 2008; Norris et al., 2005; Visser et al., 2008).

Rambling-trembling (RM-TR) decomposition of the COP has the potential to significantly improve balance deficit detection due to its proposed neurological link to postural control, segmenting the COP into an equilibrium point (RM), and oscillations around this point (TR; Ferronato and Barela, 2011; Mochizuki et al., 2006; Tahayori et al., 2012; Zatsiorsky and Duarte, 1999). RM is thought to represent the body’s equilibrium trajectory, or reference point, constantly moving and resetting, even in quiet stance. TR, on the other hand, is reminiscent of forces determined by intrinsic, pseudo-elastic musculoskeletal properties (Zatsiorsky and Duarte, 1999). Some scientists have gone as far as to attribute RM and TR components of sway to the central and peripheral nervous systems (or supraspinal and spinal), respectively (Bolbecker et al., 2018; Shin and Sosnoff, 2017; Tahayori et al., 2012). Due to these potential neuromotor links, the insights provided by RM-TR decomposition could advance our ability to identify fall risk in older adults and those suffering from somatosensory loss, but additional research is needed.

Previous work has indicated the presence of distinct sway behavior between COP, RM, and TR time series, with substantial differences in sensitivity based on severity of simulated deficit (Gerber et al., 2022). However, additional analyses are needed to further understand these time series and their empirical and clinical value in this application. One such method is nonlinear analysis, in which the system dynamics are assessed in terms of their temporal and frequency structures (Stergiou, 2016). Sample entropy (SampEn) is a commonly used nonlinear measure of human movement, describing the predictability, or regularity, of the system (Richman and Moorman, 2000). Under this framework, increasing SampEn values signify decreasing system predictability and decreasing values imply increasing predictability. This measure has shown success in its ability to distinguish healthy versus pathological conditions, as well as the influence of sensory input, and thus shows promise in fall risk assessment as it relates to sensory integration of balance (Borg and Laxaback, 2010; Ramdani et al., 2009; Roerdink et al., 2006). Therefore, this study builds upon and expands our previous study (Gerber et al., 2022), with a focused analysis of SampEn to identify COP changes driven by somatosensory input.

The objective of this study is to quantify COP, RM and TR time series using measures of range, variability (i.e., root-mean-square, RMS), and predictability (SampEn) across various levels of simulated somatosensory deficit. It is hypothesized that range, variability, and predictability will increase with foam thickness for all time series. The findings are expected to advance understanding of postural control and inform future fall risk assessment strategies.

## 2 Materials and methods

### 2.1 Participants

Fifty-two healthy young adults (aged 22.10 ± 1.88 years, 23 females) volunteered to participate in the study. All participants were informed of the study’s risks and benefits, and provided written consent, as approved by the University of Kansas Institutional Review Board. Individuals with a history of neurological disorder, balance impairments, or significant injury to the trunk or lower extremities were excluded from participation. One subject was removed from the study due to significant deviation from outcome measure means (>3σ), resulting in a final sample size of 51.

### 2.2 Experimental conditions

Participants stood naturally, with arms at the sides, eyes closed, head upright, and a standardized stance width of 17 cm and a 20° angle between feet (McIlroy and Maki, 1997). Five randomly-ordered foam thickness conditions (no foam, 1/8″, 1/4″, 1/2″, and 1″, corresponding to F0, F1, F2, F3, and F4, respectively) were used to simulate increasing severity of somatosensory deficit by decreasing the reliability of cutaneous somatosensory input, as demonstrated in the literature (Patel et al., 2008; Simoneau et al., 1995). Each foam pad was 12 inches in length and width with a density of 2 lbf/ft^3^ and pressure to compress 25% of 4 psi (McMaster-Carr, Chicago, IL). Experimental foam thicknesses were selected according to commercial availability as to maintain material property continuity and prevent potential slippage during testing. Three 60-s trials were completed for every foam condition, with 5-min seated breaks after every sixth trial.

### 2.3 Data collection and analysis

Foot-floor kinetic data was collected at 100 Hz using two 6-axis AMTI force plates (Watertown, MA, United States) and a 16-bit A/D acquisition system (Cambridge Electronic Design, Cambridge, England, United Kingdom). All data were filtered with a 2nd order 10 Hz low-pass Butterworth filter and used to calculate COP (Winter et al., 1990) using MATLAB software (Mathworks, Natick, MA).

Force and COP position trajectories were then used to calculate RM and TR time series in the AP and ML directions (Zatsiorsky and Duarte, 1999). To compute RM and TR, we first identified the time points when the horizontal ground reaction force (Fhor) crossed zero, corresponding to instant equilibrium points. The COP positions at these points were then and interpolated using a cubic spline function to form RM trajectory. The TR trajectory was subsequently derived by subtracting the RM trajectory from the original COP signal. This process was conducted in both the anteroposterior (AP) and mediolateral directions (ML).

Three primary measures were calculated: (1) range, (2) root-mean-square (RMS), and (3) sample entropy (SampEn). Based on recommendations from Nichols (2020), SampEn was calculated according to input parameters were set to m = 2 and r = 0.0986. Calculations for each measure were done independently in the AP- and ML-directions and for each level of foam thickness. Table 1 provides a convenient key to acronyms referenced throughout this work.

**TABLE 1:** Common acronyms used throughout analysis.

Acronym	Variable
AP	Anteroposterior direction (front-back)
ML	Mediolateral direction (side-side)
RMS	Root-mean-square
SampEn	Sample entropy
COP	Center-of-pressure time series
RM	Rambling time series
TR	Trembling time series
F0	Baseline foam condition (no foam)
F1	1/8″ of foam
F2	1/4″ of foam
F3	1/2″ of foam
F4	1″ of foam

### 2.4 Statistical analysis

With 95% power and an effect size of 0.25, the minimum sample size for this study was determined to be 45 participants, which was exceeded during recruitment. The repeated measure analysis of variance (ANOVA) was adopted to compare the impact of different foam thickness and on balance. Tukey’s HSD *post hoc* tests were used to determine statistical significance among foam thicknesses (F0–F4). Statistical significance for each test was set to α = 0.05.

## 3 Results

**FIGURE 1:**
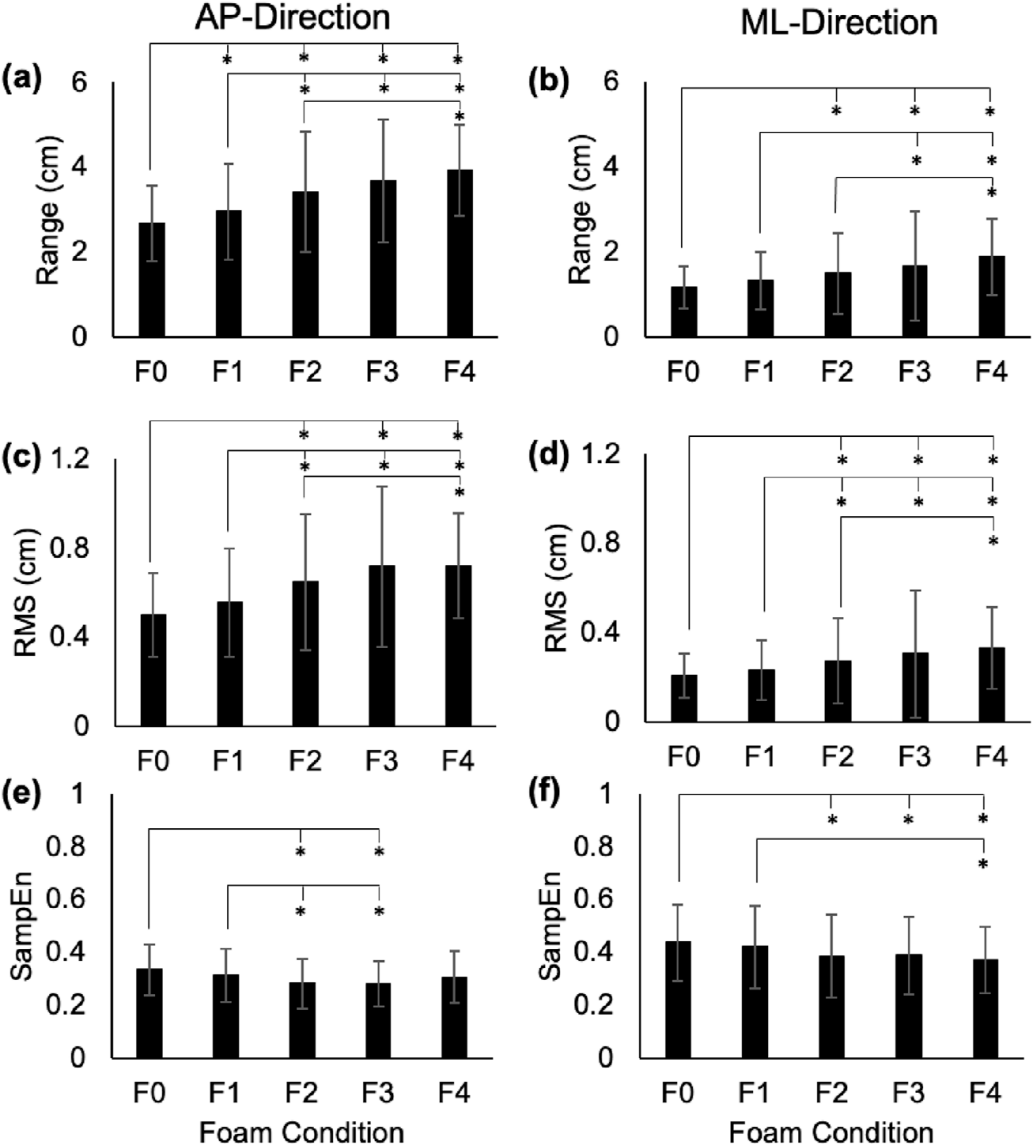
Range **(a,b)**, RMS **(c,d)**, and SampEn **(e,f)** for the COP time series in the AP and ML directions. Error bars represent standard deviations. Significant differences are shown with an asterisk (*).

**FIGURE 2:**
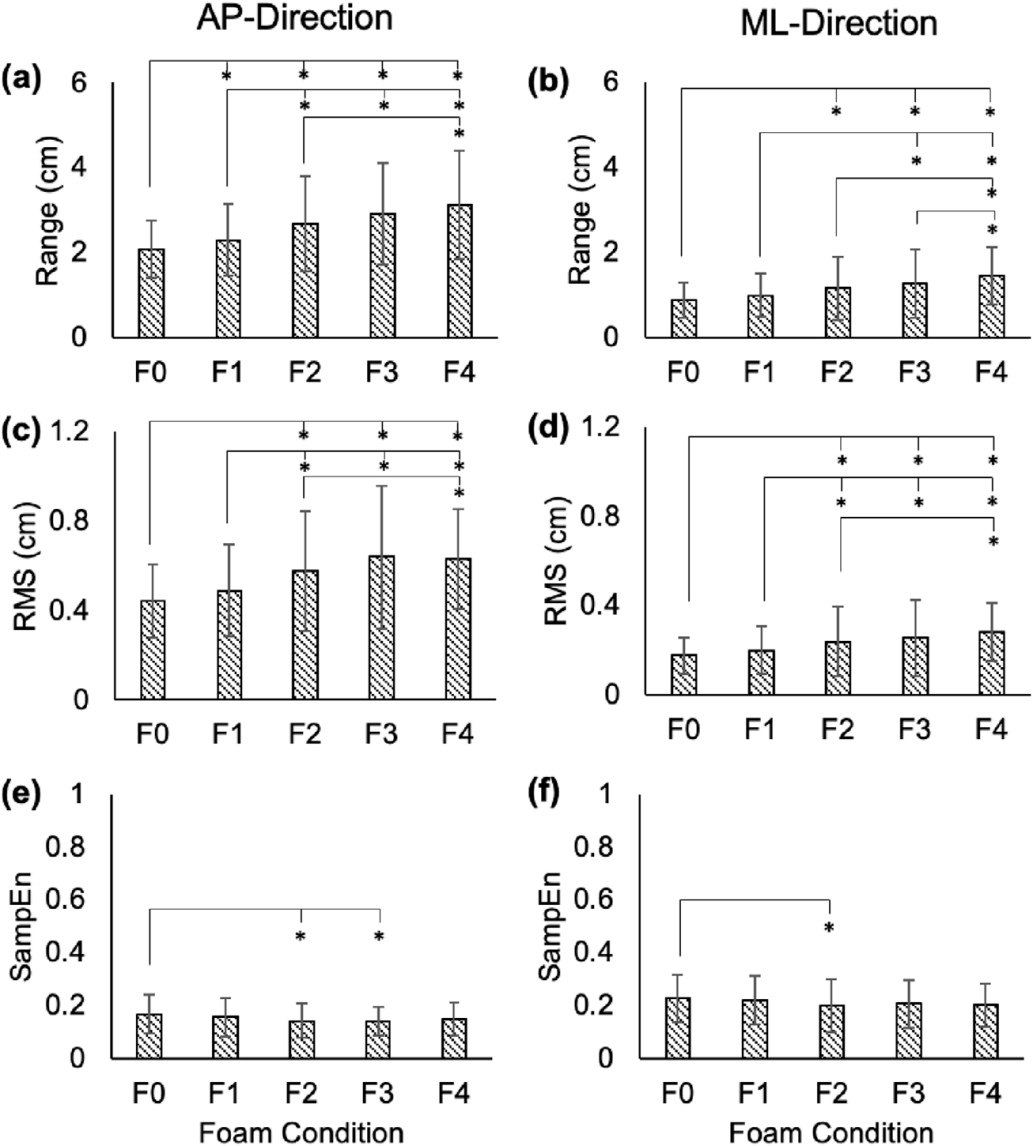
Range **(a,b)**, RMS **(c,d)**, and SampEn **(e,f)** for the RM time series in the AP and ML directions. Error bars represent standard deviations. Significant differences are shown with an asterisk (*).

**FIGURE 3:**
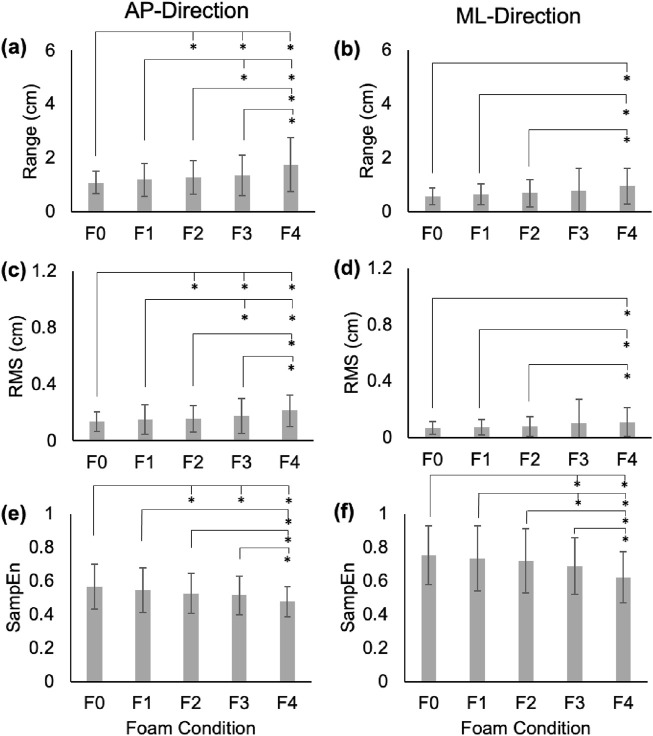
Range **(a,b)**, RMS **(c,d)**, and SampEn **(e,f)** for the TR time series in the AP and ML directions. Error bars represent standard deviations. Significant differences are shown with an asterisk (*).

Figures 1–3 depict the average range, RMS, and SampEn values for each foam condition in the AP- and ML-directions for COP, RM, and TR time series, respectively. In general, range and RMS increased across foam thickness for all time series, while SampEn decreased.

### 3.1 Range

In the AP-direction, the COP and RM time series showed significant increases between average ranges in F0 vs. F1–F4 (p < 0.01; Cohen’s d: 3.81–10.82 for COP, and 3.33–7.89 for RM), F1 vs. F2–F4 (p < 0.01; Cohen’s d: 4.76–8.06 for COP, and 5.01–6.86 for RM), and F2 vs. F4 (COP: p = 0.01, Cohen’s d = 3.4; RM: p = 0.03, Cohen’s d = 3.05; Figures 1a, 2a). In the ML-direction for COP and RM, these differences were found between F0 vs. F2–F4 (p < 0.01; Cohen’s d: 3.66–7.91 for COP, and 4.01–8.65 for RM), F1 vs. F3–F4 (p < 0.01; Cohen’s d: 3.23–8.06 for COP, and 4.05–8.79 for RM), and F2 and F4 (COP: p < 0.01, Cohen’s d = 4.04; RM: p < 0.01, Cohen’s d = 3.76; Figures 1b, 2b). For TR in the AP-direction, significant increases in range were found for F0 vs. F1-F4 (p < 0.01; Cohen’s d: 3.65–6.14), F1 vs. F3 (p = 0.02; Cohen’s d: 3.12) and F4 (p < 0.01; Cohen’s d: 6.57), F2 vs. F4 (p < 0.01; Cohen’s d: 5.45), and F3 vs. F4 (p < 0.01; Cohen’s d: 6.47; Figure 3a). In the ML-direction, TR showed significant differences between F4 and F1–F3 (p < 0.01; Cohens d: 4.46–5.94; Figure 3b).

### 3.2 Root-mean-square (RMS)

In the AP-direction, the COP and RM time series showed significant increases in average RMS values between F0 vs. F2–F4 (p < 0.01; Cohen’s d: 5.5–9.73 for COP, and 5.43–8.25 for RM), F1 vs. F2–F4 (p < 0.01; Cohen’s d: 4.31–6.47 for COP, and 4.05–5.57 for RM; Figures 1c, 2c). In the ML-direction for COP and RM, significant increases were found between F0 vs. F2–F4 (p < 0.01; Cohen’s d: 3.32–6.93 for COP, and 4.01–7.91 for RM), F1 vs. F2-F4 (p < 0.01; Cohen’s d: 2.84–7.977 for COP, and 3.1–9.09 for RM; Figures 1c, 2c), and F2 vs. F4 (p = 0.01, Cohen’s d: 3.43 for COP; p = 0.03, Cohen’s d: 2.98 for RM; Figures 1d, 2d). For TR in the AP-direction, significant differences in RMS were found between F0 vs. F2–F4 (p < 0.01; Cohens d: 2.89–8.34), F1 vs. F3–F4 (p < 0.01, Cohen’s d: 3.83–10.84), F4 vs. F2–F3 (p < 0.01, Cohen’s d: 6.39–10.34; Figure 3c). In the ML-direction, TR showed significantly different RMS values between F4 vs. F0–F2 (p < 0.01; Cohen’s d: 4.38–7.91; Figure 3d).

### 3.3 Sample entropy (SampEn)

In the AP-direction, the COP time series showed significant decreases between average SampEn values in F0 vs. F2-F3 (p < 0.01, Cohen’s d: 5.76–6.41), and F1 vs. F2–F3 (p < 0.01, Cohen’s d: 2.95–3.53; Figure 1e). In the ML-direction for the COP time series, significant decreases were found between F0 vs. F2–F4 (p < 0.01, Cohen’s d: 3.32–4.24) and F1 vs. F4 (p = 0.01, Cohen’s d: 3.48; Figure 1f). For the RM time series in the AP-direction, only F0 vs. F2–3 showed significantly different SampEn (p < 0.01, Cohen’s d: 3.56–4.15; Figure 2e). In the ML-direction, a significant decrease was found between F0 vs. F2 (p < 0.01, Cohen’s d: 2.91; Figure 2f). For TR in the AP-direction, significant differences were found between F0 vs. F2–F4 (p < 0.01, Cohen’s d: 2.93–5.63), and F4 vs. F1–3 (p < 0.02, Cohen’s d: 3.16–4.73; Figure 3e). In the ML-direction, significant differences were found between F0 vs. F3–F4 (p < 0.01, Cohen’s d: 4.28–77.84), F1 vs. F3–F4 (p < 0.01, Cohen’s d: 3.58–6.3), and F4 vs. F2–F3 (p < 0.01, Cohen’s d: 4.16–5.83; Figure 3f).

## 4 Discussion

The purpose of this study was to quantify the influence of simulated somatosensory deficit on measures of sway range, variability, and predictability. Our hypothesis was supported as the range, variability, and predictability increased with foam thickness for the COP, RM, and TR time series in both the AP and ML directions. Variability (RMS) and range showed similar increases across simulated deficit severity. Predictability was assessed using SampEn: the decrease in SampEn across foam thickness implies less systemic entropy, indicating an increase in overall signal predictability (Richman and Moorman, 2000). These findings are consistent with existing studies where foam has been shown to introduce a degree of postural instability, even in healthy young individuals, due to its viscoelastic mechanical properties and subsequent dampening of touch feedback at the plantar surface (Fujio and Takeuchi, 2021; Meyer et al., 2004; Patel et al., 2008; Perry et al., 2000). This observed effect supports the use of foam as a model for aging, with these foam-induced changes mirroring several common characteristics of sway in older adults (Degani et al., 2017; Manor and Lipsitz, 2013).

However, it is important to note that, despite mimicking many age-linked biomechanical changes to sway, the use of foam remains a rudimentary model for aging. In this study, increasing foam thickness was a simple, quantifiable means to model incrementally worsening somatosensory deficit. The overarching goal was to investigate the influence of progressive sensory loss within an individual subject, an insight nearly impossible to capture without simulated deficit (like that utilized in this study) or a multi-year longitudinal study with extensive inclusion/exclusion criteria. Though they provided many insightful learnings on the influence of somatosensation on balance, the results of this work are inherently limited in their generalizability to the elderly population. Thus, future work should incorporate a true sample of older adults with progressively worsening balance (e.g., non-fallers, history of falls, frequent fallers), using these findings to inform experimental design, analysis methodology, and interpretation.

AP and ML range in the COP, RM, and TR time series increased steadily with increasing foam thickness. In the AP-direction, COP and RM showed similar levels of significance between foam levels, presenting significant differences in as little as 1/8″ of foam thickness (F0 versus F1), but not between F3 and F4, a thickness difference of 1/2″. Conversely, TR AP range showed no significant difference between F0 and F1, but did find F3 and F4 to be different. For COP and RM, AP variability (RMS) was shown to increase across foam thickness, but F3 and F4 showed no significant differences, suggesting a plateau in this effect beyond 1/2″ of foam. Prior to F3 (1/2″ of foam), AP RMS appeared to increase incrementally with foam thickness. This observation is found also within SampEn measures. Neither COP nor RM showed significant differences in AP SampEn between F0 and F4, a comparison that, assuming linearity of simulation effect, was expected to show the highest level of contrast. Instead, COP and RM AP SampEn appear to plateau after F1, even showing a slight increase in mean values between F3 and F4. This trend is found also in RM ML SampEn, but not in COP ML SampEn, which follows a more incremental decrease across foam thickness, as expected.

It is not clear why the effects of increasing foam thickness would dissipate beyond 1/2″, but two possible explanations to this observation include (1) a ceiling effect, in which the amount of system variability is saturated, reaching a relative maximum by F3, and/or (2) given the mechanical properties of foam and high level of surface instability, the body recruits altered control mechanisms that attempt to minimize sway variability.

It is well-documented that, depending on the biomechanical challenge, the body relies on different joint strategies to maintain balance (Shumway-Cook and Woollacott, 2014). For example, Gatev et al. (1999) demonstrated that in quiet standing, the ankle is primarily responsible for postural control in the AP-direction, but reducing stance width shifts this responsibility onto the hip (Gatev et al., 1999). Fasola et al. (2019) noted that, when ankle motion was restricted, subjects heavily relied on flexion and extension of the knee to control the center of mass; in more extreme deviations, the trunk was recruited to oppose motion and correct posture (Fasola et al., 2019). Riemann et al. (2003) demonstrated that the ankle remains the primary contributor to balance on both stable and unstable support surfaces, such as foam, but noted the increase in importance of proximal joints, including the hip and knee (Riemann et al., 2003). Therefore, it is not unfounded to suggest that the observed plateau may be a result of a shift in joint-based postural control strategy at greater foam thicknesses. This would further support the use of foam as an aging model, especially at greater thicknesses, because this shift in joint strategy is also observed in older individuals, who are increasingly reliant on proximal joints (Mackey and Robinovitch, 2006). However, additional experimental methodologies, such as motion capture or electromyography would be required to support this conclusion.

The TR time series SampEn was sensitive to changes in foam thickness, presenting considerably more significant differences between foam conditions than either COP or RM. While many COP and RM measures tended to plateau beyond F3, TR SampEn scaled relatively proportionally with foam thickness, a measure characteristic that is highly desirable for tracking an individual’s balance deficit over time. Additionally, the large overall magnitude of TR SampEn suggests that, although only a small portion of overall sway, the TR time series contributes substantially to system predictability (or lack thereof). Thus, TR SampEn may serve as a powerful measure of balance deficit, especially for those suffering from somatosensory loss. This is echoed in previous work, which found ML TR to be highly sensitive to simulated somatosensory deficit, exceeding a 20% increase in maximum jerk between no foam and 1″ foam conditions, whereas COP experienced similar plateaus at greater foam thicknesses (Gerber et al., 2022). These results are expounded by findings of the present study, further highlighting the unique value that each of these time series may provide in the study of human balance and in clinical fall risk assessment.

Despite these promising findings, this study remains limited by selected outcome variables of sway range, RMS, and SampEn. These measures were carefully chosen, given their prominence in the study of aging, but there remains a wealth of unexplored sway measures and alternative methodologies, such as electromyography, that could contribute additional value to this work. Therefore, future studies should incorporate these learnings while continuing to explore a wide variety of measures to fully quantify the complex dynamic between sensation and postural control.

The study of balance is vital to both furthering our understanding of biomechanical control mechanisms and to improving fall risk assessment techniques. Somatosensory decline poses a significant risk to the aging population, reducing the accessibility of critical environmental and proprioceptive cues. The findings of this study highlight the scientific value of rambling-trembling methodology, examining sway from a mechanistic perspective and providing new, clinically-relevant insights into postural control. Though there is much work to be done to fully comprehend the utility of rambling-trembling, it shows tremendous promise in its ability to identify and track the progression of somatosensory deficit.

Lastly, though it is not a perfect replication of decreased rapidly adapting mechanoreceptive sensation, as highlighted by Patel et al. (2011), standing on foam is widely used in both research and clinical settings as a practical and controlled method to challenge somatosensory input and reduce the reliability of proprioceptive feedback from the plantar surface and ankle joints (e.g., Diener et al., 1984; Shumway-Cook and Horak, 1986). The use of varying foam thicknesses, as we proposed, is intended to create a graded reduction in somatosensory reliability, rather than to fully replicate a specific sensory deficit. This degradation also provides a useful proxy for studying the effects of sensory challenge on postural control.

## Data Availability

The original contributions presented in the study are included in the article/supplementary material, further inquiries can be directed to the corresponding author.
